# Physical Activity Intervention for Loneliness (PAIL) in community-dwelling older adults: protocol for a feasibility study

**DOI:** 10.1186/s40814-018-0379-0

**Published:** 2018-12-19

**Authors:** Anastasia V. Shvedko, Janice L. Thompson, Carolyn A. Greig, Anna C. Whittaker

**Affiliations:** 1 School of Sport, Exercise and Rehabilitation Sciences, University of Birmingham, Birmingham, UK; 2 MRC – Arthritis Research UK Centre for Musculoskeletal Ageing Research, University of Birmingham, Birmingham, UK

**Keywords:** Feasibility study, Physical activity, Loneliness, Older adults, Randomised controlled trial

## Abstract

**Background:**

Low-quality social relationships in older adults are strongly associated with feelings of loneliness. Physical activity interventions could reduce loneliness and improve psychological well-being, among other health benefits. The aim of this study is to examine the feasibility of a Physical Activity Intervention for Loneliness (PAIL) in community-dwelling older adults at risk for loneliness.

**Methods/design:**

This feasibility study is a two-arm randomised controlled trial (RCT) with a wait-list control group using a mixed-methods research design. The primary aim of the feasibility study is to estimate recruitment, retention and adherence rates; the appropriateness of the intervention design and its practicality; the acceptability of the intervention by participants; and the set of instruments and measures and primary outcome measures to inform a future large-scale randomised trial. After eligibility screening, randomisation will be conducted using computer-based random sequence generation. Baseline and post-intervention assessments for intervention and control groups will include height, weight, body mass index, resting blood pressure, physical activity using accelerometry, loneliness, social support, social networks, anxiety and depression, self-efficacy for exercise, satisfaction with social contacts, and expected outcomes and barriers for exercise using questionnaires. Focus groups will be conducted at the mid-point and post-intervention period using a phenomenological approach to analyse the participants’ experiences of taking part in PAIL.

**Discussion:**

This trial will provide important information regarding the feasibility of PAIL in community-dwelling older adults at risk for loneliness using a mixed-methods approach combining quantitative and qualitative research methods.

**Trial registration:**

Clinicaltrials.gov NCT03458793

**Electronic supplementary material:**

The online version of this article (10.1186/s40814-018-0379-0) contains supplementary material, which is available to authorized users.

## Background

Maintenance of social connectedness throughout the lifespan is an important aspect of successful ageing [1]. The disruption of established social patterns or poor quality of social relationships negatively impacts quality of life and well-being in older adults and is highly associated with loneliness [2, 3]. According to UK statistics, loneliness is highly prevalent and increasing among older adults aged 65 years or older [4]. Further, 11% of people aged 75 years and older reported to have no close friends and visited their general practitioner (GP) to fulfil their need to talk to somebody [4]. Defined as a discrepancy between a person’s desired and actual social relationships [5], loneliness and a lack of social relations were considered to be high-risk factors for morbidity and mortality, and the negative impact of loneliness can be as harmful as smoking 15 cigarettes a day [6, 7]. Bearing in mind the ageing of the UK population, with a sharp increase in the proportion of adults aged 65 years and older over the last 30 years [8], health professionals have placed special emphasis on the promotion of “active ageing” enabling older adults to increase participation in “social, economic, cultural, spiritual and civic affairs” to maintain quality of life [9]. Further, promotion of a variety of campaigns to prevent loneliness [10–12] may reduce or slow the burden on NHS expenses, with potential economic benefits estimated at around £900 per annum per person associated with loneliness reduction [10]. Given this, the early prevention of loneliness and timely implementation of health interventions in a community setting to tackle the problem at its early onset seems prudent.

Physical activity (PA) is defined as any bodily movement produced by the contraction of skeletal muscles that results in a substantial increase over resting energy expenditure [13, 14]. It is a health behaviour that can facilitate meaningful social relationships and serve as an alternative to medical treatments which may have negative side effects [11, 15, 16]. Compared to other forms of treatment therapies (e.g. mindfulness therapy, art and craft therapy), physical activity interventions, especially in small groups (up to eight to nine people), can assist in building friendly and trusted relationships between participants based on shared interests and similar needs, as demonstrated by previous research [12, 17].

Mechanisms of physical activity interventions’ effectiveness are suggested to relate to loneliness reduction models, stress reduction and increased social support during activities. Related to the first mechanism, the social compensation model [18] suggests that PA can work via compensation for lost meaningful social connections due to increased peripheral social networking during friendly conversations between participants [17]. The hypothesis of the broaden-and-build theory of positive emotions [19] posits that enjoyable forms of PA generate happiness and bring positive emotions, which in turn could be associated with loneliness reduction as shown in a longitudinal study Newall et al. [20]. The tripartite model of group identification was found to be effective, particularly among lonely seniors, based on the sense of identification and social attraction to group members with shared interests and goals, arising during engagement in physical activities [21]. Related to the second mechanism, based on the stress/social support model [22], social networks promote well-being that is associated with loneliness reduction in older adults. Among the mechanisms named above, only the tripartite model of group identification was shown to be successfully applied in the treatment of loneliness directly in the context of PA.

Based on the analysis of existing randomised controlled trials (RCTs), there are few PA interventions for loneliness reduction conducted with residents in community settings [23]. This is also in line with previous systematic reviews and meta-analyses [2, 15, 24–30]. A subsequent analysis for other loneliness-related social outcomes has shown that for social functioning (as a sub-domain of health-related quality of life), specific aspects of PA interventions can successfully influence social health [23] with the strongest effects being obtained for group setting exercise interventions, with delivery by a health/medical professional, in a diseased rather than healthy population. PA interventions did not appear to be effective for increasing social support or social networks [23]. In addition, the majority of studies used a cross-sectional design, which does not allow determination of causality and limits the rigour of the research evidence. However, longitudinal studies do not always support the direct effects of PA interventions on loneliness reduction in older adults [15]. Nevertheless, it is important to emphasise that the majority of existing PA interventions assess loneliness as a secondary outcome within a number of other psychosocial outcomes, which limits the ability to fully examine these interventions’ effectiveness for reducing loneliness [15, 31].

Further, a number of moderating (affecting the strength of the relationship between PA and loneliness) and mediating factors (driving the influence of PA on loneliness) are not consistently considered in intervention studies, making the outcomes of even well-conducted interventions less credible [15]. Research shows that global- [32, 33] and domain-specific self-efficacy [34] are moderating factors, and social support was found to be both a moderating [35] and mediating factor [36]. Also, perceived self-efficacy in one study by Fry and Debats [34] was found to be a superior predictor of loneliness and psychological distress in older adults compared to demographic factors, physical health and support networks. Further, personal (self-efficacy) and environmental (social support) variables moderating relationships between loneliness and PA have a bidirectional link with each other [37]. Perceived social support is a moderator of self-efficacy, and increased self-efficacy leads to better social support [35].

Bearing in mind the limitations of the current literature as presented above, understanding the mechanisms of association between loneliness and PA may bring new insights to the designing of novel and effective PA interventions [15]. Further research is needed to explore the association between loneliness, self-efficacy and social support in the context of PA interventions for older adults. However, before the mechanisms can be fully understood, the practicalities and feasibility of implementation of such interventions with older adults must be tested. The aim of the study is an examination of the feasibility of RCT of Physical Activity Intervention for Loneliness (PAIL) in community-dwelling older adults at risk for loneliness. For the planned future large-scale RCT, the primary hypothesis is that, compared with the inactive control group, participants in the intervention group will report a greater decrease in loneliness. The secondary hypothesis is that participants in the intervention group will significantly increase their amount of physical activity engagement per week, and this will be associated with greater positive changes in other psychosocial and health outcomes compared to control group participants.

### Study objectives

The following specific aims of this feasibility study are to estimate:

1. Recruitment rate, attendance and retention rates (number of participants completing the study as a proportion of those randomised)
2. The appropriateness and practicality of the designed intervention in the proposed settings
3. The acceptability of the intervention by participants and willingness to participate
4. The appropriateness of the assessment tools
5. The appropriateness of the statistical methods of data analysis used
6. The power calculation of the likely required sample size for a future large-scale RCT.
7. The acceptability of measures and the most suitable primary outcome measure for a future large-scale RCT

In addition, to reflect the aims of a future large-scale RCT that this feasibility study seeks to inform, the effect sizes between the intervention and control groups will be examined.

## Methods/design

PAIL is a tw0-arm RCT of a 12-week intervention compared with a wait-list control (WLC) condition using a mixed-methods research design (including quantitative and qualitative research methods). Quantitative data will be collected from the intervention and control groups at the baseline and immediate post-intervention periods. Qualitative data aimed to assess the appropriateness, practicality and acceptability of the designed intervention in the proposed settings will be collected at the mid-point and post-intervention period. The trial was approved by the Science, Technology, Engineering and Mathematics (STEM) Research Ethics Committee of the University of Birmingham, UK (ERN_16-1419A). Consent forms (Additional file 1) will be obtained from all participants prior to entry into the study. This protocol for a feasibility study was guided by the SPIRIT 2013 Checklist (Additional file 2).

### Eligibility

Participants will be recruited from local neighbourhoods (households) and community centres across the wide geographic area of Birmingham, UK.

#### Inclusion criteria

The following are the inclusion criteria:

1. Community-dwelling older adults aged 60 years and older;
2. Previously sedentary (i.e. as defined by a lack of regular involvement in more than 20 min exercise per week over the past month that increased breathing significantly and was considered moderate) [38];
3. At risk of loneliness and having ≥ 6 out of 9 points on the three-item loneliness scale during the phone screening [39] (Additional file 3);
4. Physically mobile as measured using the Short Physical Performance Battery (SPPB) [40] with a score ≥ 9 out of 12 [41];
5. Healthy or having one or more common chronic diseases but ambulatory, without a cognitive disability as assessed by the Montreal Cognitive Assessment (MOCA) [42] with a score ≥ 22 out of 30 [43];
6. Able to give written informed consent;
7. English speaking and able to complete paper and pencil questionnaires.

#### Exclusion criteria

The following are the exclusion criteria:

1. < 60 years old;
2. Currently taking part in another physical activity intervention;
3. Socially active or not lonely based on the phone screening tool by Hughes et al. [39];
4. Regularly physically active;
5. Moderate to severe cognitive disability with cut-off below 22 for MOCA or clinical diagnosis of dementia or Alzheimer’s disease;
6. Not ambulatory, i.e. not able to walk 4 m;
7. Not literate in English (speaking and reading) as this precludes taking pen and paper tests.

### Interventions

The PAIL feasibility study is a 12-week intervention consisting of group walking and health educational/social interaction workshops performed once weekly for a duration of up to 90 min per session. The design and features of the PAIL intervention are based on the features of effective interventions that were obtained from a systematic review and meta-analysis of the existing evidence conducted by Shvedko et al. [23]. Effective interventions were physical activity (PA) rather than PA interventions with social interactions, organised in group settings versus individually or one-to-one, delivered by a medical healthcare provider versus non-qualified providers and with strongest effects obtained for diseased versus healthy populations.

Group walking sessions will be run once weekly for up to 45 min each in small groups (up to eight to nine people per group) and delivered by a trained walk leader (i.e. level 3 certified personal trainer and a group exercise instructor). Prior to the first walking session, participants in the intervention group will be asked to complete the “Par-Q and You form” [44] and will receive a copy of general practitioner (GP) letter that informs their GP about their participation in the study. Participants attending the walking intervention will join the walking leader following a specified route in varied locations at every session to maintain interest in the intervention [45]. During guided walking, the instructor will be acting as a facilitator of social contact by using in-session talks and friendly discussion between participants to reduce psychosocial tension. Walking sessions will be based on the principles of gradual progression and adaptation to PA [13]. The intensity of the walks will be monitored objectively by heart rate monitors using the age-predicted heart rate maximum (HR_max_) method [46] by calculating in advance the age-predicted zones of heart rate intensity using the formula 220 minus age in years. Additionally, the intensity of walks will be monitored subjectively using the 0–10 Borg Ratings of Perceived Exertion (RPE) Scale [47] and the talk test [48]. Participants will be provided with a standardised set of instructions about the use of the 0–10 Borg RPE Scale [47, 49] and talk test [48] to enhance their understanding of these methods before the intervention [13]. Light-to-moderate intensity walking will be monitored by the ability of participants to talk back comfortably during exercises using the talk test [48] and 2–4 on the 0–10 Borg RPE scale [47]. A warm-up will be performed at the beginning of each session for 7–10 min and will include preparatory dynamic and static stretches standing, performed for the major muscle groups and walking at a leisurely pace maintaining an upright pose. At the end of the walk, participants will perform up to 10 min of balance training recommended for older adults to reduce frailty [50]. A further cool down will be performed at the end of the walking session for 5–7 min and will consist of stretching exercises for upper and lower body performed in two regimes: maintenance (e.g. maintenance of the muscle length) and developmental stretches (e.g. involving the gradual lengthening of a muscle group into an elongated position and subsequent “hold” of this position). At the end of the cool-down, participants will perform breathing exercises while standing for about 2 min.

Group walking sessions will be followed by the health education/social interactions workshops delivered in the form of a group presentation weekly for up to 45 min by the researcher (PhD student) on a variety of healthy ageing topics such as eye hygiene, mental health and well-being, preventing falls, social support, nutritional guidelines and physical activity recommendations for older adults (Additional file 4).

#### Intervention group

After randomisation, participants in the intervention group will start the 12-week walking intervention.

#### WLC group

Participants in the wait-list control (delayed intervention) group will start the intervention after their follow-up measures are completed approximately 12 weeks post-randomisation.

### Measures

**Fig. 1:**
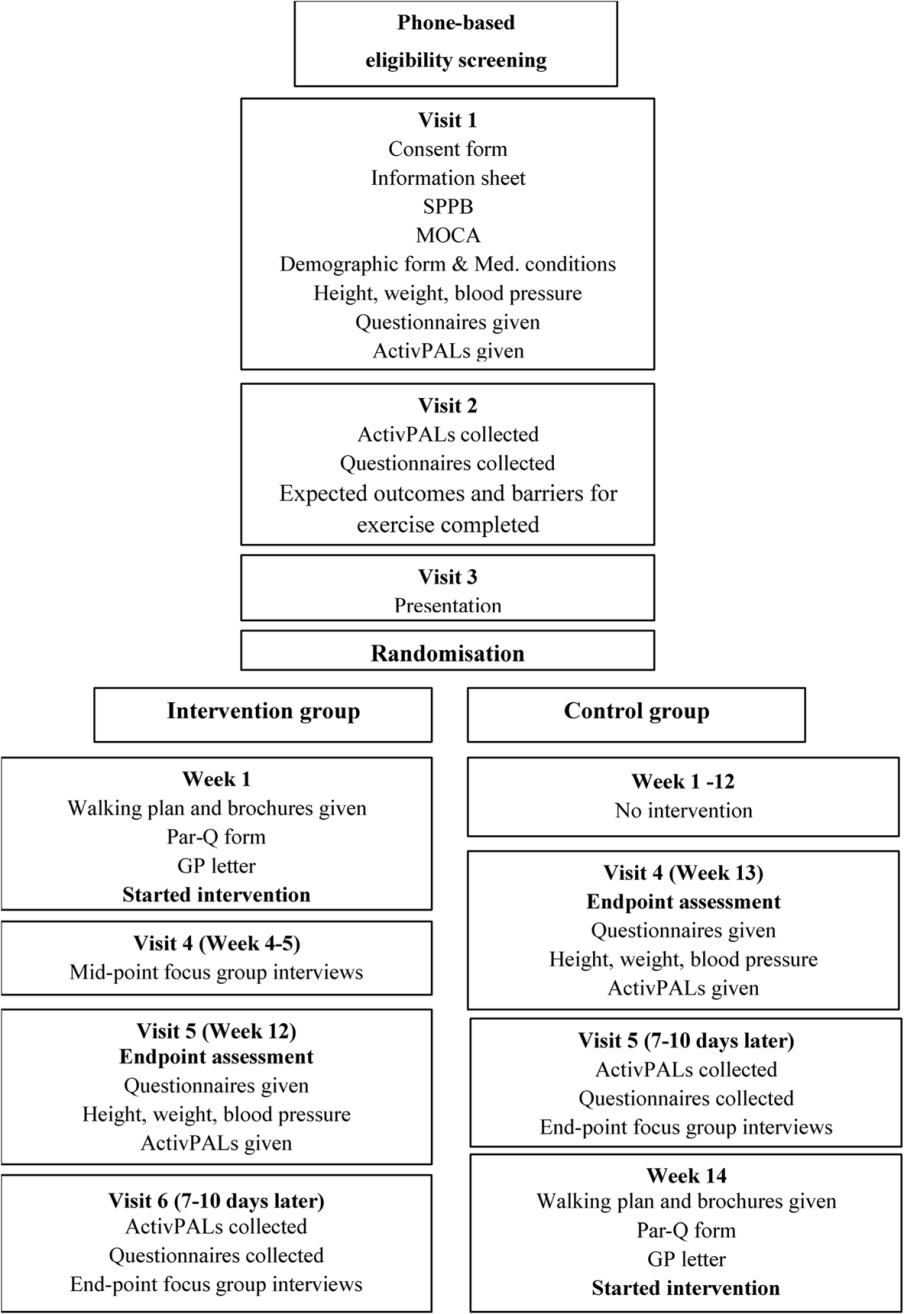
Chart of study visits

All measures will be conducted at the host academic institution at baseline and at the immediate post-intervention period (Fig. 1) except for focus groups, which will take place at the mid-point and post-intervention.

#### Socio-demographic characteristics and medical conditions

Participants will provide socio-demographic information including their age, gender, ethnicity, marital status, living arrangements, level of education, any children, employment status and any medical conditions.

#### Health measures

*Cognitive function* will be assessed using a Montreal Cognitive Assessment (MOCA) scale designed to test mild cognitive impairment [42]. *Physical functioning* will be assessed using the Short Physical Performance Battery (SPPB) [40]. *Height* to the nearest 0.1 cm will be measured using a stadiometer (Seca AG, Reinach, Switzerland) and recorded in metres. *Weight* will be assessed using weighing scales (Tanita UK Ltd., Middlesex, UK) to the nearest 0.1 kg. *Body mass index* (BMI, kg m^−2^) will be calculated by dividing mass (kg) by height squared (m^2^). *Resting blood pressure* (BP rest, mmHg) will be measured using a portable semi-automatic OMRON sphygmomanometer (OMRON HEM705CP sphygmomanometer; Omron Matsusaka Co Ltd., Japan). *Physical activity* will be measured using ActivPAL accelerometers (PAL Technologies Ltd. Glasgow, UK) at baseline and immediate post-intervention period over a continuous 7-day period of awake and sleeping (24 h a day) except when bathing or swimming [51].

#### Questionnaires

*Loneliness* will be assessed using the 8-item UCLA Loneliness Scale (UCLA-8) [52]. *Social support* will be assessed using the 20-item Medical Outcomes Study Social Support Survey (MOSSSS) [53]. *Social networks* will be assessed using the 6-item Lubben’s Social Network Scale (LSNS-6) [54]. *Depression and anxiety* will be assessed using the 14-item Hospital Anxiety and Depression Scale (HADS) [55]. *Self-efficacy for exercise* will be measured using the revised 9-item Self-Efficacy for Walking/Exercise Scale (SEE) using a paper-and-pencil format [56]. *Satisfaction with level of social contacts* will be measured with the question “How satisfied are you with your social contacts?” [57]. *Expected outcomes and barriers for exercise* will be administered using the Expected Outcomes and Barriers for Habitual Exercise scale [58] adapted for the older adult population. Four questions related to sport competence have been deleted from the expected outcomes sub-scale due to irrelevance for this population group [58]. The expected outcomes and barriers for exercise scale have demonstrated good internal consistency from 0.66 to 0.85 and a high test-retest reliability of 0.78 in previous research [58].

#### Qualitative assessments

To understand participants’ experiences of taking part in the PAIL feasibility trial, focus groups will be conducted at mid-point (between the weeks 4 and 5) and at the end of the 12-week intervention using semi-structured discussions in small groups (four to nine people per group) of mixed gender (Additional files 5 and 6). The research team will check if any alterations will be required based on the participants’ feedback. The 32-item checklist for reporting qualitative research will guide the researcher to ensure clarity and transparency in focus groups methodology [59]. Focus groups will be audio recorded using a digital recorder and will be transcribed verbatim. An independent trained focus group leader will act as a moderator and facilitator of the focus groups [60].

#### Feasibility outcomes

The following feasibility outcomes will be assessed in this study:

1. Attendance will be calculated as the total number of attended sessions divided by the total number of sessions of the intervention and recorded as a percentage;
2. Recruitment rate will be calculated as the number of individuals responding to advertisements and friends’ referrals out of a total number of formal invitations given/advertisements placed (including web-based advertisements, advertisements placed in the local cohort groups and poster and leaflet material disseminated in the community). Recruitment rate will be recorded as a percentage, e.g. 25% (48/195). It is acknowledged that advertisements may reach a large number of individuals but it is impossible to quantify this;
3. Retention rate will be calculated as the number of participants completing the study as a proportion of those randomised;
4. The appropriateness, practicality and acceptability of the designed intervention in the proposed settings will be assessed using focus group interviews. The focus group transcripts will be analysed using a phenomenological inductive approach [61], and these data will be used to help the research team to improve the quality of the delivered intervention by informing positive changes in the methodology and design of the intervention for the future implementation in a consequent study;
5. The assessment rate of questionnaires will be evaluated as the total number of completed questionnaires divided by the total number of questionnaires and recorded as a percentage;
6. The appropriateness of statistical methods of data analysis will be analysed by the research team in terms of suitability to the data;
7. A power calculation and sample size estimation will be calculated for meaningful potential future primary outcomes (e.g. loneliness or social support) using a method based on the differences in means between the intervention and control groups, using the G-power software [62];
8. The effect size (ES) will be calculated for loneliness, social support, social networks, anxiety and depression, self-efficacy for exercise, satisfaction with level of social contacts and the expected outcomes and barriers for exercise. Means (M) and standard deviations (SD) will be used to investigate the effect size for change in loneliness using mixed between (intervention group vs. control group) and within (over time) repeated-measures analysis of variance (ANOVAs) with post-hoc comparisons.

### Data monitoring

The data monitoring committee for this project is the supervisory research team (three academic supervisors) who are independent of the trial sponsor. They will be responsible for checking data accuracy upon assembly of the final database following completion of data collection prior to data analysis. The PhD student is responsible for monitoring and reporting spontaneous adverse events or any unintended trial effects to the supervisory team, and the PI (Whittaker) is responsible for reporting these to the sponsor. The trial is also subject to independent audit request by the sponsor, the University of Birmingham, by a team independent to the supervisory/research team. The sponsor contact details are given below.

Dr Sean Jennings, Head of Research Governance, University of Birmingham (e-mail: s.jennings@bham.ac.uk).

### Data collection

Data will be collected at the university facility at screening, baseline and post-intervention period (12 weeks after the start of the intervention) (Fig. 1). After providing baseline eligibility screening, potential participants will be offered a total of five visits for health assessments at the university facility. Participants in the intervention group will have an additional sixth visit for attending the mid-point focus group. All digital data (transcripts, digital information, health assessment databases) will be stored securely on the password-protected university computers available only to the research team. All other data (e.g. eligibility screening forms, activity data and questionnaire data) will be pseudo-anonymised with a unique ID number and stored in locked filing cabinets/on password-protected university computers accessible only to the research team. Only the researchers will have access to the data.

### Sample size

As this will be the feasibility study to inform the design of the future large-scale RCT, a total target sample of 40 older adult participants will be recruited for estimation of the critical parameters [63] with 20 in the intervention group and 20 in the wait-list control group.

### Recruitment

Participants will be recruited from local neighbourhoods (households) and communities in Birmingham via a two-step strategy. First, through widespread advertisement in local media resources (newspaper, University of Birmingham website), via posters (Additional file 7) and distribution of leaflets about the study in local community centres and shops, churches, temples and mosques, local libraries, post offices, veterinary surgeries; and through advertisements via the University of Birmingham’s 1000 Elders Group [64], and Ageing Better Consortium BVSC [65]. This is to recruit an ethnically diverse sample that is reflective of the Birmingham population. Secondly, recruitment will be facilitated during the eligibility screening where potential participants will be given a copy of the information sheet (Additional file 8) or a leaflet about the study and will be asked to invite anyone else they know and who might be interested in the project, i.e. using snowball or chain recruitment by word of mouth [66].

### Randomisation and concealment

Randomisation will be conducted after completion of baseline measures using a computer-generated random sequence performed by an external researcher not involved in the delivery of the intervention or outcome assessment. Participants will be informed about the group allocation by e-mail or a phone call by a person not involved in assessments or delivery of the intervention. At the outcome assessment level, participants who will be assessors of their own psychosocial outcomes using questionnaires will be blinded to their group allocation at the time of completing the initial questionnaires. Intervention providers who will be responsible for outcome assessments will not be blinded to the intervention delivery as this would not be possible, given that the study and walks will be conducted by the PhD student (AS) who is the researcher.

### Recruitment and retention rates

Recruitment will be aimed to be at a rate of 10 participants a month (to a minimum of 40 participants) for estimation of the critical parameters of the feasibility study [63]. If the number of recruited participants is less than 75% by the end of the 4-month recruitment period or if the retention rate is less than 75% at 12 weeks (end-point period), changes will be made to the recruitment strategy and the intervention will run again a few months later. No targets were set for other feasibility outcomes, e.g. questionnaire completion rates or attendance at the intervention sessions.

### Progression criteria

As suggested by El-Kotob et al. [67], feasibility studies are not adequately powered to test the hypothesis of the efficacy of physical activity intervention, and therefore, a priori criteria for progression to the definitive large-scale RCT is advisable to consider future efforts. The progression criteria to a definitive large-scale RCT were (1) no any serious adverse events, such as hospitalisation, life-threatening condition, death and any adverse events associated with the intervention experienced by less than 5% of participants per group; (2) recruitment rate of no less than 75% by the end of the 4-month recruitment period; and (3) retention rate of no less than 75% in each group at 12 weeks (end-point). If all the three criteria were not met, there would be insufficient evidence to justify proceeding to the definitive RCT. Changes will be required to be made to the intervention with the consequent re-running of the intervention a few months later.

### Statistical methods

#### Quantitative data analysis

Quantitative data will be analysed using SPSS version 22.0 for Windows (SPSS Inc., Chicago, IL) employing an intention-to-treat analysis (based on their treatment allocation and irrespective of participants’ adherence or withdrawal) [68]. The level of significance will be set at *p* < .05; however, any hypothesis testing is preliminary and results will be interpreted with caution as this pilot study is underpowered and the analyses based on small numbers. Baseline differences between groups for continuous data (e.g. age, BMI, resting blood pressure, number of comorbidities, cognitive and physical functioning, and outcomes of questionnaires) will be analysed using one-way ANOVA. Chi-squared tests will be applied for nominal data (e.g. gender, ethnicity, marital status, living arrangements, level of education, children and employment status). For descriptive statistics, data will be presented as means (M) and standard deviations (SD). Nominal data will be presented as number (*N*) and percentage. Mixed between (group) and within (time) repeated-measures ANOVAs with post-hoc comparisons will be applied to investigate the effect of the intervention versus control on psychosocial outcomes (loneliness, social support, support networks, depression, anxiety, self-efficacy for exercise, satisfaction with level of social contacts), expected outcomes and barriers for exercise, and accelerometer data. The accelerometer data will be analysed using the ActivPAL software V7.1.18 (PAL technologies, Scotland, UK). Recorded data will be downloaded to a computer, and data for average daily amount of stepping (step counts), average time lying and sitting (h) in increments of 15 s, average time standing (h) and energy expenditure (EE, MET/h) will be analysed using mixed between (intervention group) and within (time) ANOVAs. For the Expected Outcomes and Barriers for Habitual Exercise scale [58], additional test-retest reliability will be calculated via correlation. In order to explore which outcome measures are likely to be the most important for the main trial, Pearson’s correlations will be performed between calculated change scores over time in the experimental group for all psychosocial outcomes (Lubben’s social networks, loneliness and self-efficacy for exercise) and change scores for averaged daily physical activity (time lying/sitting (h), time standing (h), time stepping (h), step counts, sit-to-stand transitions (n) and energy equivalent (METs/h)). A power calculation and sample size estimation for a future large-scale RCT will be calculated for meaningful outcomes (e.g. loneliness or social support) using the method based on the differences in means between the intervention and control group using the G-power software Version 3.1 [62].

#### Qualitative data analysis

Qualitative data will be thematically analysed by two research team members independently using a phenomenological inductive approach [61]. Transcripts will be returned to participants for comments/correction to ensure transparency and trustworthiness of the data (member checking) [69]. Validated transcripts will be read several times by two independent researchers to obtain an overall meaning. Then, themes and subthemes with important meanings common for all participants will be derived from the obtained data. Results will be compared through discussion between reviewers [70].

### Data storage and protection

Research data will be kept for 10 years in line with UK data protection regulations [71]. Physical data will be pseudo-anonymised with a unique ID number and stored confidentially in locked filing cabinets/on password-protected university computers accessible only to the research team. Digitally recorded interview transcripts will be stored securely on a password-protected computer that only the researchers will have access to. Audio recordings will be destroyed after the recordings are transcribed verbatim.

## Discussion

This study will explore the feasibility and acceptability of the Physical Activity Intervention for Loneliness (PAIL) in community-dwelling older adults. The key features of the study are based on effective components of physical activity interventions to treat loneliness obtained from a systematic review and meta-analysis of the existing evidence that highlighted the lack of physical activity interventions for older adults residing in community settings [23]. Studies to date either included non-PA interventions only or mixed design studies for only one or two outcomes (e.g. RCTs, case-control studies, longitudinal). The randomised controlled design of this feasibility study allows improvement upon the methodological rigour of the current evidence. Consequently, this feasibility study uses objective measures of PA and comprehensive methods of assessment of social health which will allow estimation of the mechanisms of association between PA and social health outcomes in older adults in the future large-scale study. Findings from this study will be of the interest to healthcare professionals and community organisations working with older adults at risk of loneliness or social isolation in the implementation of exercise interventions to address this problem in society.

### Dissemination

Dissemination will include a summary of the results for participants, publication of an open-access article detailing the full findings (also part of the PhD student’s thesis) and presentation of study findings at conferences. The results of this feasibility study will be used to inform the development of a large-scale RCT. Participants in the intervention will be sent a feedback information sheet with the results of the study by e-mail or post (if no e-mail is available). The findings are also intended to be disseminated at the British Geriatric Society Loneliness annual meetings and via news at www.campaigntoendloneliness.org. The published study protocol and journal paper will be uploaded to the Current Research Information System (CRIS) – PURE portal available at pure.bham.ac.uk open to the academic staff, students and external researchers.

### Trial status

This feasibility trial is registered at Clinicaltrials.gov Identifier: NCT03458793. Ethical approval for the project was received from the STEM Research Ethics Committee of the University of Birmingham, UK (ERN_16-1419A). Participant recruitment and baseline data collection started in October 2017 and is expected to be finished by November 2018. The study will be fully completed by May 2019.

## Additional files

Additional file 1: Consent form (DOCX 61 kb)

Additional file 2: The SPIRIT 2013 Checklist (DOCX 122 kb)

Additional file 3: Phone-based eligibility screening form (DOCX 47 kb)

Additional file 4: Content of group workshops (DOCX 17 kb)

Additional file 5: Mid-point focus group questions (DOCX 24 kb)

Additional file 6: End-point focus group questions (DOCX 18 kb)

Additional file 7: Information sheet (DOCX 282 kb)

Additional file 8: Recruitment poster (DOCX 359 kb)

## Abbreviations

ANOVA: Repeated-measures analysis of variance
BMI: Body mass index
BP rest: Resting blood pressure
BVSC: Birmingham Voluntary Service Council
EE: Energy expenditure
ES: Effect size
GP: General practitioner
HADS: Hospital Anxiety and Depression Scale
HR_max_: Heart rate maximum
LSNS-6: 6-Item Lubben’s Social Network Scale
M: Means
MET: Metabolic equivalent
MOCA: Montreal Cognitive Assessment
MOSSSS: Medical Outcomes Study Social Support Survey
*N*: Number
PA: Physical activity
PAIL: Physical Activity Intervention for Loneliness
RCT: Randomised controlled trial
RPE: Borg’s Ratings of Perceived Exertions
SD: Standard deviations
SEE: Self-efficacy for exercise scale
SPPB: Short Physical Performance Battery
STEM Ethical Review Committee: Science, Technology, Engineering and Mathematics Ethical Review Committee
UCLA-8: 8-Item The University of California, Los Angeles Loneliness Scale
WLC group: Wait-list control group
