# Supplementary material for: Physical Activity Intervention for Loneliness (PAIL) in community-dwelling older adults: protocol for a feasibility study
Source: Pilot Feasibility Stud. 2018 Dec 19;4:187. doi: 10.1186/s40814-018-0379-0 (PMC6299531; doi:10.1186/s40814-018-0379-0)
Supplement: Supplementary file 1 — Consent form (DOCX 61 kb) [file 40814_2018_379_MOESM1_ESM.docx]

| **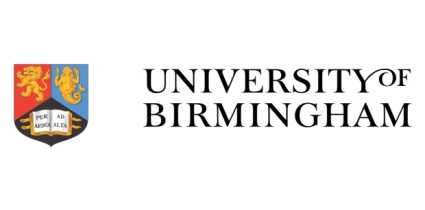** | **Additional file 1** Consent form |
| --- | --- |

**Participant’s Consent Form *[Office use only: ID ______]***

**Physical Activity Intervention for Loneliness (PAIL) in community-dwelling older adults: A feasibility study**

Please read the following statements and sign if you agree to take part in the study.

| I have read the information sheet (version 1, 12.4.17) concerning this study and understand what it is about. All my questions have been answered to my satisfaction. I understand that I am free to request further information at any stage. Please initial each box.   \| 1. My participation in the study is entirely voluntary. \|  \| \| --- \| --- \| \| 2. I agree to participate and consent to my data being used and stored against a unique ID number for 10 years \|  \| \| 3. I understand I will be required to answer questions about my feelings. \|  \| \| 4. I understand that I can withdraw from the study at any time without giving reason, but that any research data will still be used unless I request otherwise. \|  \| \| 5. I know that the information that I give will be treated as confidential and my name will not be used in any publications. \|  \| \| 6. I know that I may ask that any data pertaining to me be destroyed at any time up until research publication with no penalty. \|  \| \| 7. I agree to my GP being informed that I am taking part in this study \|  \| \| 8. I am happy to be contacted about future research. \|  \|   I agree to the above statements. |
| --- | --- | --- | --- | --- | --- | --- | --- | --- | --- | --- | --- | --- | --- | --- | --- | --- |

Signed

.……………………………………………………

Print name

……………………………………………………. Date ……………………

The study has been reviewed and approved the Ethics Committee of the University of Birmingham. Thank you for your time and consideration.

| Anastasia Shvedko  Doctoral Researcher  School of Sport, Exercise & Rehabilitation Sciences  axs1235@student.bham.ac.uk  Tel: 44 (7) 490 392 720 | Prof Anna C Whittaker  Principal Investigator  School of Sport, Exercise & Rehabilitation Sciences  [A.C.Whittaker@bham.ac.uk](mailto:A.C.Whittaker@bham.ac.uk)  Tel. 44 121 414 4398 |
| --- | --- |
