# Supplementary material for: Physical Activity Intervention for Loneliness (PAIL) in community-dwelling older adults: protocol for a feasibility study
Source: Pilot Feasibility Stud. 2018 Dec 19;4:187. doi: 10.1186/s40814-018-0379-0 (PMC6299531; doi:10.1186/s40814-018-0379-0)
Supplement: Supplementary file 3 — Phone-based eligibility screening form (DOCX 47 kb) [file 40814_2018_379_MOESM3_ESM.docx]

**Additional file 3** Phone-based eligibility screening

**Physical Activity Intervention for Loneliness (PAIL) in community-dwelling older adults: Participant Screening**

Hello, thank you for showing an interest in the walking programme for adults. My name is Anastasia and I am a Doctoral Research student at the University of Birmingham.

I am doing a research on the impact of physical activity on well-being and health of older adults. As a part of my project, you are invited to take part in the 12 week outdoor group walking with a trained walk leader and a social club over a cup of tea. This is an opportunity for you to increase your physical activity and make new friends. The programme is free of charge and will be held at variety of locations around the Edgbaston campus based on the preferences and time that we arrange. The full information will be given to you later that is provided in the information sheet, so you can take it with you and share with your friends or family.

To take part, first, we need to check if you fit the study during the phone-based screening that will only takes **10 minutes of your time**.

If you fit then we will invite you for a second eligibility testing that will check your ability to walk and your cognitive abilities, such as logic, memory and a language.

We will need to have your verbal consent to the telephone-based screening.

Do you have any questions before we proceed?

Would you like to start our phone assessment? Yes No

We would need to take your contact information:

| Name |  | | | | | | | | | | | | |
| --- | --- | --- | --- | --- | --- | --- | --- | --- | --- | --- | --- | --- | --- |
| Telephone |  | | | | | | | | | | | | |
| E-mail |  | | | | | | | | | | | | |
| Date of Screening: | | | | | | | | | | | | | |
| Verbal consent for telephone screening given | | | | | | | | Yes | | | | | No |
| Gender | | | | | | | | Male | | | | | Female |
| How did you hear about **the PAIL**? | | 1. Advertisement  2. By word of mouth (i.e. referred by a friend)  3. Other (please state)  __________________________________________ | | | | | | | | | | | |
| *“Now I’m going to run through a series of questions and when we get to the end I’ll explain whether you could be eligible to take part in PAIL study and if you are we’ll go onto a few more questions.”* | | | | | | | | Yes | | | No | | |
| **A. Eligibility criteria** | | | | | | | |  | | |  | | |
| 1. Are you 60 years old or over? | | | | | | | | Yes | | | No | | |
| 2. Do you live in the community residence (e.g. private housing)? | | | | | | | | Yes | | | No | | |
| 3. Have you been diagnosed by your GP with Alzheimer’s disease or dementia? | | | | | | | | Yes | | | No | | |
| 4. Are you currently enrolled in another physical activity research project or physical activity intervention? **If yes:**  Study name: ______________________________________  Study end date:____________________________________ | | | | | | | | Yes | | | No | | |
| **Did the participant fall into any of the shaded boxes, or involved in any other physical activity intervention or research study?** | | | | | | | | Yes  ⏷ | | | | No | |
| **If ‘Yes’, explain that:**  *“Unfortunately, we can’t include you in the intervention PAIL at the moment because you are not eligible for the participation in the study, but if you are happy for us to do so, we will contact you to inform about any other opportunities in the future”.* | | | | | | | | | | | | | |
| *If “****No”*** *you may proceed with the eligibility screening.* | | | | | | | | | | | | | |
| **B. Assessment of feeling socially isolated or lonely (3-item tool of Hughes et al., 2004 for the phone screening)** | | | | | | | | | | | | | |
| *The next questions are about how you feel about different aspects of your life.*  *For each one, tell me how often you feel that way using responses: “Hardly ever”, “Some of the time” or “Often”.* | | | | | | | | | | | | | |
| 5. First, how often do you feel that you lack companionship: “Hardly ever”, “Some of the time”, or “Often”? | | | Hardly ever  1 | | Some of the time  2 | | | | Often  3 | | | | |
| 6. How often do you feel left out: “Hardly ever”, “Some of the time”, or “Often”? | | | Hardly ever  1 | | Some of the time  2 | | | | Often  3 | | | | |
| 7. How often do you feel isolated from others? (Is it “Hardly ever”, “Some of the time”, or “Often”?) | | | Hardly ever  1 | | Some of the time  2 | | | | Often  3 | | | | |
| Provide **the total scoring** **for questions 5-7** by the sum of answers. | | | Total sum of points __________________ | | | | | | | | | | |
| *If* the loneliness score is ***≥6 points*** *the participant is at risk of feeling socially isolated or lonely and is* ***eligible*** *to participate. You can proceed with the screening and may go* ***to section C****.* | | | | | | | | | | | | | |
| **If No explain that:**  *“Unfortunately, we can’t include you in the intervention PAIL at the moment because you are not eligible for the participation in the study, but if you are happy for us to do so, we will contact you to inform about any other opportunities in the future”.* | | | | | | | | | | | | | |
| **C. Assessment of physical function** | | | | | | | | | | | | | |
| *Now I will ask you questions related to your health”* | | | | | | | | | | | | | |
| 17. Do you have any chronic illness or a medical condition? | | | | | | | Yes | | | No | | | |
| 18. If you answered **yes** on previous question, are you physically mobile and able to walk without support? | | | | | | | Yes | | | No | | | |
| “*A few more questions related to your health…”* | | | | | | | | | | | | | |
| 19. A) Do you use a wheelchair? | | | Yes | | | | | | | | | | No |
| B) Do you use a Zimmer frame? | | | Yes | | | | | | | | | | No |
| 20. How would you find walking across a room? *(With a walking stick is OK. If needs a zimmer* ***tick ”Very difficult”)*** | | | Easy  ⏷ | A little difficult | | Very difficult | | | | | | | Unable |
| 21. How easy would you find getting out of a low chair? | | | Easy  ⏷ | A little difficult | | Very difficult | | | | | | | Unable |
| a) If response is “Easy” ask: Would you normally use your arms to help you get up from the chair? | | | Yes / No | | | | | | | | | | |
| 22. How easy would you find walking up a flight of stairs with no handrail or wall to lean on? | | | Easy  ⏷ | A little difficult | | Very difficult | | | | | | | Unable |
| 23. How easy do you find walking on an uneven pavement without losing your balance? | | | Easy  ⏷ | A little difficult | | Very difficult | | | | | | | Unable |
| **Do any of the participant’s answers fall into any of the shaded boxes, or they find all four activities very difficult or unable?** | | | | | | Yes  ⏷ | | | | | | | No |
| **If ‘yes’, explain that:**  *“Unfortunately, we can’t include you in the PAIL trial at the moment because your level of physical function is not appropriate for the participation in the study, but if you are happy for us to do so, we will contact you again in a few months to re-assess the situation.”* | | | | | | | | | | | | | |
| *If* ***No*** *participant is eligible to take part in the study and you may proceed with the eligibility screening* | | | | | | | | | | | | | |
| **Date to call back (time and date) if needed:** _________________________________________ | | | | | | | | | | | | | |

| **D. Assessment of physical activity and exercise behaviour** | | | | | | | | |  |
| --- | --- | --- | --- | --- | --- | --- | --- | --- | --- |
| *“I will just continue to ask a few more questions about your level of physical activity and participation in any exercises”* | | | | | | | | |  |
| 24. Can you remember, during the past 4 weeks (past month), did you do any regular exercise **over 20 minutes/week?** | | | | | Yes | | | No |  |
| If the answer is **NO** go to section E**.** Participant is eligible to take part in the study.  If the answer is **YES** proceed asking more question and go to question 25. | | | | | | | | |  |
| 25. Can you remember, in a typical or normal week during the past 4 weeks, did you spend at least 20 minutes a week getting regular exercise **that increased breathing significantly and considered to be moderate, I will give you examples:** (*Circle the appropriate answer below*). | | | | | | | | |  |
| 1. Dance? (for example, square, folk, line, ballroom) (*Interviewer Note:* ***Do not count aerobic dance****)* | | | | Yes | ­­­_____  mins | | | ______  times/week |  |
| 1. Walk uphill or hike uphill? | | | | Yes | _____  mins | | | ______  times/week |  |
| 1. Walk fast or briskly for exercise? | | | | Yes | _____  mins | | | ______  times/week |  |
| 1. Water exercises? (*Such as aqua aerobics NOT leisure swimming)* | | | | Yes | _____  mins | | | ______  times/week |  |
| 1. Other _____________________   (if this exercise significantly increased breathing. You can talk but you can not sing the words). | | | | Yes | _____  mins | | | ______  times/week |  |
| ***If any YES answer:*** *“Can you say how much time did you do this exercise and how often did you do this exercise a week for the past four weeks? (write in columns above)?” (refer to the section above and fill the answers)* | | | | | | | | |  |
| *Sum of all items (a,b,c,d,e)* | | | | _________________  Total minutes a week | | | | |  |
| ***Total sum is* ≥ 125 minutes/week *for the past four weeks (past month)?*** | | | | Yes | | | No | |  |
| If the answer is **YES explain** that “unfortunately you seem to be active at the moment and you are ineligible to participate in the study”. Would you like us to call you back in four weeks’ time?  Due to call back (date and time) ______________________________________ | | | | | | | | |  |
| **If answer is NO** participant is eligible to take part in the study. **Go to section E.** | | | | | | | | |  |
| **E. Confidence in ability to get to sessions:**  *“None of us can predict exactly what will happen over the next year, holidays, illnesses and minor operations may crop up unexpectedly, but in general if you were allocated to the physical activity sessions how confident do you feel that you would usually be able to attend a PAIL session once a week for the next three months.”* | | | | | | | | |  |
| Quite confident | Not sure | | Not at all confident | | | | | | |
| If not at all confident ask and record the reason(s) why:  ___________________________________________________________________  **If NOT confident because of the location of the venue or days of the week explain:** *“Other venues/days will become available later this year, which may be more convenient for you. Would you like us to call you back then?*  Due to call back (date and time) **___________________________**  **If still not confident, suggest that:** *“Would it be better if we called you back in a few months to see if you are feeling more capable of attending sessions?”* | | | | | | | | |  |
| Agreed to be called back  Date and time : _________________ | | Preferred not to be called back | | | | | | | |
| *Thank you. That’s all I have to ask you at the moment, is there anything you’d like to ask me?”............................. “From what you have told me so far you could be eligible to take part in our study so what we would like to do now is invite you to attend a session at (venue)__________ so that we can meet face to face and conduct a few more simple screening tests such as asking you to walk 4 metres and answer a few more questions.”* | | | | | | | | |  |
| **Are you still happy to attend?** | | | Yes | | | No | | |  |
| **If ‘No’: “***We will be running PAIL in other areas around the city later this year. Would you prefer to be put on the waiting list for these other sessions?* | | | Yes | | | No | | |  |
| **If ‘Yes’, set date and time of the call:** ___________________________________ | | | | | | | | |  |
| **If still ‘No’:** *‘That is not a problem at all, if you change your mind, please do get back in touch with us by telephone, or e-mail.* | | | | | | | | |  |

**Explain further details of the screening session (on the next page).**

*“It’s been nice talking to you. That’s it for today. If there are any more questions you want to ask you can call me anytime. Thank you very much for your time. Preliminary screening shows that you are eligible to take part in our study.*

*At the next stage of the screening we need to arrange the meeting with you to check your cognitive skills and physical mobility. We can do it at home or in our facility”.*

| *At University facility* |  | |
| --- | --- | --- |
| *At home* |  | |
| Date | Time | Address |
|  |  |  |
|  |  |  |
|  |  |  |
|  |  |  |

*Researcher:-* □ Enter details onto database Date ____________________
