# Supplementary material for: Physical Activity Intervention for Loneliness (PAIL) in community-dwelling older adults: protocol for a feasibility study
Source: Pilot Feasibility Stud. 2018 Dec 19;4:187. doi: 10.1186/s40814-018-0379-0 (PMC6299531; doi:10.1186/s40814-018-0379-0)
Supplement: Supplementary file 4 — Content of group workshops (DOCX 17 kb) [file 40814_2018_379_MOESM4_ESM.docx]

**Additional file 4** Content of group workshops

**Content of group workshops**

1. Healthy ageing
2. Healthy eating: nutritional guidelines for older adults
3. Loneliness and social isolation
4. Social support
5. Physical activity recommendations for older adults
6. Local social events and exercise activities
7. The importance of the ‘flu vaccination
8. Mental health and well-being
9. Dental hygiene
10. Eye hygiene
11. Keep active
12. Preventing falls
