# Supplementary material for: Physical Activity Intervention for Loneliness (PAIL) in community-dwelling older adults: protocol for a feasibility study
Source: Pilot Feasibility Stud. 2018 Dec 19;4:187. doi: 10.1186/s40814-018-0379-0 (PMC6299531; doi:10.1186/s40814-018-0379-0)
Supplement: Supplementary file 5 — Mid-point focus group questions (DOCX 24 kb) [file 40814_2018_379_MOESM5_ESM.docx]

**Additional file 5** Mid-point focus group questions

**Mid-point focus group questions**

Date:

Place:

*Hello, my name is …. I am going to lead a focus group with you today. I will be asking you questions about the PAIL intervention, about your experience and attitudes of taking part in the study. Please be honest. Your opinion is valuable to us and helps us to improve our research in the future.*

*You had participated in the 4/12 week walking interventions and attended healthy workshops. Tell us a bit about your experience so far/ of taking part in the PAIL study*.

| Was there any particular reason or goal you had which led you to participate in this intervention? |  |
| --- | --- |
| Tell us about your experience of participation in the intervention. What part of the sessions did you enjoy most? What did you like about it? |  |
| What part of the sessions did you least enjoy and why? |  |
| Do you feel the instructor was well prepared for walking intervention and for the group discussions? |  |
| Do you think that the content of the social group workshops was relevant/useful to you and what would you suggest to include/exclude in the future? |  |
| Were you happy with the total length of the sessions? |  |
| If you feel that intervention has to be changed how you would improve it? |  |
| Was there anything that could have been done differently, including preparing equipment or the facility to improve your experience? |  |
| Tell us your opinion about the questionnaires used in the study. Did you find easy to use them? Did you experience any specific difficulties? |  |
| How did you find the activity monitors? Did you have any problems with wearing them? Is there anything you would like to suggest? |  |
| Did you have any difficulties in attending the course regularly? |  |
| Did you have any personal barriers to participation in the intervention regularly? |  |
| Was the research team supportive to you and helped you throughout the intervention? Was there anything that disturbed you? |  |
| Do you think that if you regularly take part in this intervention you will be able to make new friends? |  |
| We would like to know how do you get on with people in the group so fare. At what session of the intervention did each of you start to engage in the conversation with others or you feel that you need more time? |  |
| What has been the most useful or meaningful experience for you during the intervention and what do you feel you will take away from this experience? |  |
| Is there any more information you would like about what you need to do to reach your desired physical activity goals or make the sessions more interesting to you? |  |
| How else could the sessions have been improved for you? |  |

*Thank you for your time. Your opinion is very important to us. Your answers will be confidential and your name will not be mentioned anywhere where direct quotes are published in order to illustrate this research study.*
