# Supplementary material for: Physical Activity Intervention for Loneliness (PAIL) in community-dwelling older adults: protocol for a feasibility study
Source: Pilot Feasibility Stud. 2018 Dec 19;4:187. doi: 10.1186/s40814-018-0379-0 (PMC6299531; doi:10.1186/s40814-018-0379-0)
Supplement: Supplementary file 6 — End-point focus group questions (DOCX 18 kb) [file 40814_2018_379_MOESM6_ESM.docx]

**Appendix 6** End-point focus group questions

**Endpoint focus group questions**

*Hello, my name is ……. I am going to ask you few questions about the PAIL intervention you have just taken part in. Please, don’t think too much and say what comes first to your mind. Your immediate response is more important. Are you ready, so let’s get started?*

*You had participated in the12 week PAIL walking intervention and attended healthy workshops. Tell us a bit about your experience*.

1. What have you learnt from the intervention?

2. Did you have any difficulties during the intervention?

3. What things have helped you to overcome any difficulties?

4. Do you think you made new friends? If yes, how many and at what session did you start feeling this?

5. What has been the most meaningful experience for you and what do you feel you will take away from this experience?

*This is the end of the interview. Thank you for your time. This is very important for us. Your answers will be confidential and your name will not be mentioned anywhere.*
