# Supplementary material for: Physical Activity Intervention for Loneliness (PAIL) in community-dwelling older adults: protocol for a feasibility study
Source: Pilot Feasibility Stud. 2018 Dec 19;4:187. doi: 10.1186/s40814-018-0379-0 (PMC6299531; doi:10.1186/s40814-018-0379-0)
Supplement: Supplementary file 7 — Information sheet (DOCX 282 kb) [file 40814_2018_379_MOESM7_ESM.docx]

| **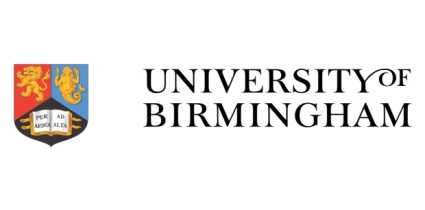** | **Additional file 7** Information sheet |
| --- | --- |

**Physical Activity Intervention for**

**Loneliness (PAIL) in community-dwelling older adults: a feasibility study**

**INFORMATION SHEET**

Investigators: Prof Anna Whittaker, Dr Carolyn Greig, Prof Janice Thompson , Doctoral Research Student Anastasia Shvedko (University of Birmingham).

You are being invited to take part in the current study that is looking to see if it is possible to do a group walk and health education workshops to increase levels of physical activity in older adults residing in the community. This information sheet explains purposes of the study, the reasons why you may be asked to take part and conditions of your participation. Please, take the time to read this information sheet and decide if you wish to consent to take part in the study.

**1. What is the purpose of the study?**

This study will address the following aims:

1. To assess the feasibility and practicality of the Physical Activity Intervention for Loneliness (PAIL) in community-dwelling older adults.

2. To assess the effect of the PAIL on loneliness reduction.

3. To analyse the associations between loneliness, self-efficacy for exercise and social support in terms of the impact of the PAIL.

**2. Who are we looking for?**

You have been invited because you are:

- Community-dwelling older adult aged 60 years or over;
- Inactive (i.e. engaged in less than 20 minutes per week of regular physical activity for the past month or <125 minutes per week of moderate physical activity). Moderate intensity aerobic exercise is where you are working hard enough to raise your heart rate and break into a sweat (e.g. activity like walking at a fast pace). You are able to talk but unable to sing the words to a song;
- You may sometimes feel lonely or socially isolated;
- Physically mobile and able to walk without support (walking stick is acceptable);
- Healthy or having one or more common chronic diseases but mobile;
- Without a cognitive disability;
- English speaking and able to complete paper and pencil questionnaires.

**3. What does this study include?**

You will take part in the 12-week outdoor group walking with the trained walking leader (Doctoral Research Student of the University of Birmingham) and will listen to a series of health education workshops. During walks you will be following the walking leader by the specified route starting from the University of Birmingham facility. You will receive the walking plan and walking brochures to facilitate exercise activity in your free time.

**4. Do I have to take part?**

- Your participation in the study is entirely voluntary.
- You can withdraw from the study at any time without giving a reason. However, we would still like to use any data you have provided by this point, unless you specifically request for it to be destroyed.
- You are guaranteed the security and confidentiality of data collected during research.

**5. What will happen if you decide to take part?**

**If eligible, you will** be scheduled a meeting at your preferred location (at home or in the University of Birmingham facility) for you to give initial consent to the study and to obtain information about your health and physical mobility at **Visits 1 and 2**. You also will be asked to complete a demographic information form and a medical conditions you may have using the form.

**If eligible to proceed,** you will be invited to group meeting in the University of Birmingham facility (**Visit 3**) to listen to the presentation about the study and meet other participants over a cup of tea. You will be asked to complete self-reported questionnaires to assess your feelings. We will also measure your height, weight and resting blood pressure and give you an activity monitor (small gadget to be worn on the thigh and secured with waterproof dressing) to measure your physical activity over a continuous 7 day period (24 hours a day) except for bathing/swimming (showering is okay) (Picture 1).

**Pic. 1.** Activity monitor.

We will also inform you if we find any results during the study which are of clinical significance.

**Focus group interviews**

Focus group interviews will be conducted in small groups (up to 9 people in the group) of mixed gender twice between week 4 and 5 and at the end of the 12 week course using semi-structured discussions. The duration of a single interview is approximately 1 hour (range 1-1.5 hours). In the group you will be asked to discuss a series of open-ended questions over tea and biscuits with the trained focus group leader. The aim of focus groups will be to

gather your experiences of taking part in the study, overall content and delivery of the course, and other questions to help the research team to improve the study in the future based on your honest feedback.

**6.** **What are the possible disadvantages and risks of taking part?**

There are minimal risks to taking part In the PAIL study. During the guided walking of a moderate intensity you may feel tired or out of breath, but this is normal and safe.

**7. What are the potential benefits of taking part in the study?**

Your feedback on the study will be used to make some alterations and design an effective study in the future to increase physical activity and prevent loneliness and social isolation in older adults. You will also receive a summary of the study findings upon completion of the 12 week programme.

**8.** **What will happen if I do not want to carry on with the study?**

You can withdraw up to 2 weeks after final session. During the study, you can withdrawal at any time without needing to give a reason by contacting the research team. You will be offered to fill up the withdrawal form to us to identify the possible reasons for your withdrawal and asked if you would like to be contacted in the future.

**9. What will happen to the results of the research study?**

The results of the study will be a part of the dissertation research project of the postgraduate student and will be published in the journal paper. Authors of the study guarantee the anonymity in any published information; names of participants will not be given. Where opinions stated in the focus groups are quoted in publications, we will change names to preserve anonymity. Research data will be stored labelled with a unique ID number in locked filing cabinets/on password protected university computers accessible

only to the research team and only the researchers will have access to information linking you to your unique ID number in order to contact you to

arrange the visits during the study. Research data will be kept for 10 years in line with UK data protection regulations.

**10. Who has reviewed this study?**

The study has been reviewed and approved by the Ethics Committee of the University of Birmingham.

**11. What happens now if I decide to take part?**

If you wish to participate, please contact Anastasia Shvedko on **07490392720** or e-mail to [axs1235@bham.ac.uk](mailto:axs1235@bham.ac.uk). You will be arranged the date and the time for the phone-based eligibility screening for the study participation which takes only 15 minutes. We will be glad to answer any questions you have about the study.

**This information sheet is yours to take home.**

**Thank you for your time. It is appreciated.**

| Anastasia Shvedko  Doctoral Researcher  School of Sport, Exercise &  Rehabilitation Sciences  axs1235@bham.ac.uk  Mob: 44 (7) 490 392 720 | Prof Anna Whittaker  Principal Investigator  School of Sport, Exercise &  Rehabilitation Sciences  [A.C.Whittaker@bham.ac.uk](mailto:A.C.Whittaker@bham.ac.uk)  Tel. 44 121 414 4398 |
| --- | --- |
