# Supplementary material for: Physical Activity Intervention for Loneliness (PAIL) in community-dwelling older adults: protocol for a feasibility study
Source: Pilot Feasibility Stud. 2018 Dec 19;4:187. doi: 10.1186/s40814-018-0379-0 (PMC6299531; doi:10.1186/s40814-018-0379-0)

**
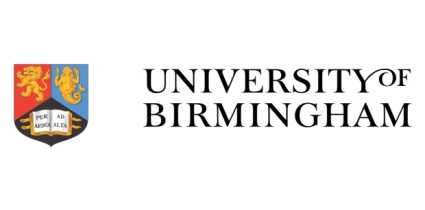
****AA**

**Additional file 8** Recruitment Poster


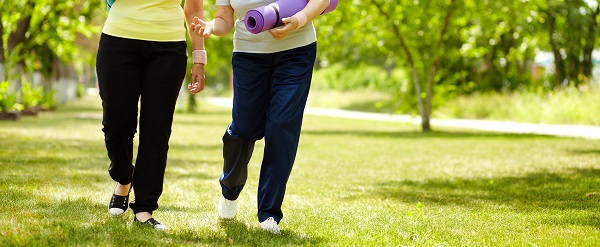


**Aged 60 and over?**

**Looking for friends?**

Need some exercise?

We would like to invite you to the

**12 WEEK OUTDOOR GROUP WALKING PROGRAMME**

Organised by doctoral research students from the University of Birmingham, UK

The programme is free of charge. You will be invited to attend once weekly walking group sessions with the trained walk leader. As a part of the course you will attend healthy workshops for older adults and a social club discussion over a cup of tea. This is an opportunity for you to make new friends, increase your physical activity and have a good time.

**WHO ARE WE LOOKING FOR?**

- Older adults aged 60 years or over;
- Inactive (less than 20 minutes of a regular exercise per week for the past month);
- At risk of loneliness or social isolation (e.g. living alone, recently widowed, with a long-term care responsibility, etc.);
- Able to walk without support (walking stick is acceptable);
- Healthy or having one or more common chronic diseases but ambulatory;
- English speaking and able to complete paper and pencil questionnaires.

**WHAT DO I HAVE TO DO?**

Health assessments usually take about 30-minutes and include a total of 5 visits for assessment of height, weight, resting blood pressure, physical activity, **questionnaires** and **two focus group interviews.**

For more information, please call at **07490392720** or e-mail: [axs1235@bham.ac.uk](mailto:axs1235@bham.ac.uk) (Doctoral research student Anastasia Shvedko)


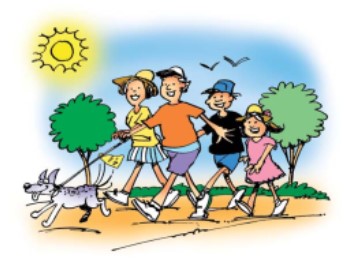

Supplement: Supplementary file 8 — Recruitment poster (DOCX 359 kb) [file 40814_2018_379_MOESM8_ESM.docx]
